# Delaying disease progression in COPD with early escalation to triple therapy: a modelling study (DEPICT-2)

**DOI:** 10.1183/23120541.00438-2024

**Published:** 2025-04-07

**Authors:** Dave Singh, Diego Fabian Litewka, Joan B. Soriano, Adrian Rendon, Frederico Leon Arrabal Fernandes, Rafael Páramo-Arroyo, Tim Trinidad, Hakan Günen, Sudeep Acharya, Bhumika Aggarwal, Gur Levy, Chris Compton, Abdelkader El Hasnaoui, Peter Daley-Yates

**Affiliations:** 1 Division of Immunology, Immunity to Infection and Respiratory Medicine, The University of Manchester and Manchester University NHS Foundation Trust, Manchester, UK; 2 Unidad Neumonologia, Hospital Juan A. Fernandez, Buenos Aires, Argentina; 3 Servicio de Neumología, Hospital Universitario de la Princesa, Facultad de Medicina, Universidad Autónoma de Madrid, Centro de Investigación Biomédica en Red de Enfermedades Respiratorias, Instituto de Salud Carlos III, Madrid, Spain; 4 Universidad Autónoma de Nuevo Leon, Hospital Universitario “Dr Jose Eleuterio Gonzalez”, CIPTIR, Monterrey, Mexico; 5 Disciplina de Pneumologia, Instituto do Coração, Hospital das Clínicas, Faculdade de Medicina, Universidade de São Paulo, São Paulo, Brazil; 6 Universidad Anáhuac Querétaro, Centro de Estudios Clinicos de Querétaro, Querétaro, Mexico; 7 University of Santo Tomas, Faculty of Medicine and Surgery, Manila, Philippines; 8 Health Sciences University, Süreyyapaşa Research and Training Center for Chest Diseases and Thoracic Surgery, Istanbul, Turkey; 9 Emerging Markets, GlaxoSmithKline, Singapore; 10 Emerging Markets, GSK, Panama City, Panama; 11 GSK, Brentford, UK; 12 Emerging Markets, GSK, Dubai, United Arab Emirates; 13 Independent Clinical Pharmacology Consultant, London, UK

## Abstract

**Introduction:**

In patients with COPD, dual bronchodilator (long-acting muscarinic antagonist (LAMA)/long-acting β2-agonist (LABA)) and triple therapy (inhaled corticosteroid/LAMA/LABA) reduce the risk of exacerbations and lung function decline in the short–mid-term, but their long-term impact is unknown. This modelling study explores long-term impact of these therapies on lung function decline, quality of life (QoL) and all-cause mortality.

**Methods:**

This modelling approach used a longitudinal nonparametric superposition model using published data regarding exacerbations, QoL (assessed by St George's Respiratory Questionnaire (SGRQ)) and mortality. The model simulated disease progression from 40 to 75 years of age and assessed the impact of initiating dual bronchodilator at age 45 years (“LAMA/LABA only” group) and escalation to triple therapy at age 50 years (“Escalation to triple” group) on forced expiratory volume in 1 s (FEV_1_) decline, QoL and mortality.

**Results:**

Model simulation predicted that by 75 years of age, “LAMA/LABA only” preserves 159.1 mL of FEV_1_ *versus* no treatment, while “Escalation to triple” preserves an additional 376.5 mL and 217.3 mL of FEV_1_ *versus* no pharmacotherapy and “LAMA/LABA only”, respectively. In “LAMA/LABA only”, the SGRQ score reduces (−3.2) *versus* no treatment, which further reduces to −7.5 in “Escalation to triple”. In “LAMA/LABA only”, mortality reduces by 5.4% by 75 years *versus* no treatment, while the “Escalation to triple” shows further decrease in mortality by 12.0%.

**Conclusion:**

Early pharmacotherapy initiation and escalation from dual bronchodilator to triple therapy could slow disease progression by preserving lung function and improving QoL and survival in patients with COPD.

## Shareable abstract

**DEPICT-2, a modelling study investigating long-term outcomes for pharmacotherapy in COPD, found that early escalation to ICS + dual bronchodilator triple therapy from dual bronchodilators may result in improved lung function and QoL, and reduced mortality** https://bit.ly/3XjoND5

## Introduction

COPD is characterised by progressive lung function decline [1, 2], which tends to be more rapid in mild-to-moderate COPD compared with severe COPD [1]. The rate of decline is also more rapid in patients experiencing frequent exacerbations compared with nonexacerbating patients, and in smokers compared with nonsmokers [3]. There are many consequences of exacerbations, including increased risk of future exacerbations and mortality, as well as a decrease in quality of life (QoL) and accelerated lung function decline [4, 5].

The main goals of COPD management are to reduce symptom burden, improve health status, prevent exacerbations, reduce lung function decline and reduce risk of mortality [1]. While bronchodilator(s) improve respiratory symptoms, randomised controlled trials (RCTs), such as IMPACT and ETHOS, have shown that inhaled corticosteroid (ICS)-based triple therapies reduce the risk of exacerbation and decrease the risk of mortality compared with dual bronchodilator in patients with history of exacerbations [6, 7]. The timing of initiation of pharmacotherapy also plays a key role as early initiation may result in exacerbation reduction, reduction in the rate of decline of lung function and improvement in QoL, compared with pharmacotherapy initiated when the disease has become more severe [8, 9].

Most current clinical trials in COPD have much shorter follow-up times; hence, clinical data spanning the lifetime duration of disease (≥30 years) are not available. As the long-term effects of initiation of pharmacotherapy in the early stages of the disease course are difficult to evaluate in a clinical setting, a modelling approach was utilised in the initial DEPICT study (DElaying Disease Progression In COPD with Early Initiation of Dual Bronchodilator or Triple Inhaled PharmacoTherapy (DEPICT): A Predictive Modelling Approach) [10]. The study explored the effects of dual and triple therapies initiated at different time points during the course of the disease on lung function decline, and demonstrated the benefits associated with early intervention, especially with triple therapy, in reducing the loss of lung function [10]. The DEPICT study modelled the effects of triple therapy at an early stage of the disease, despite current practice and recommendations suggesting that triple therapy is introduced as an escalation from dual bronchodilators, mainly in patients who have already experienced exacerbations. Moreover, DEPICT did not evaluate the effects of early pharmacotherapy on other parameters, such as QoL and survival [10].

In this DEPICT-2 study, through a modelling approach, we aimed to simulate the long-term benefits of treatment initiation with dual bronchodilators at 45 years of age and the effects of escalation from dual bronchodilator to triple therapy at 50 years of age on lung function decline (forced expiratory volume in 1 s (FEV_1_)), QoL (St George's Respiratory Questionnaire (SGRQ)) and all-cause mortality in COPD.

## Methods

### Model structure and input

The modelling approach utilised the data extracted from published literature to construct a longitudinal nonparametric superposition model. A detailed model structure has been presented previously [10]. For baseline assumptions, a comprehensive targeted literature search was conducted using PubMed and Google Scholar databases to identify relevant studies on lung function (FEV_1_), QoL (SGRQ) and mortality in COPD. Search terms specified the topics of interest. Further relevant data were identified through grey literature search. Studies presenting data from RCTs, pooled analyses, retrospective studies and real-world observational studies conducted in COPD patients were included.

The targeted search topics included:

1) Impact of COPD severity on lung function decline, QoL and mortality.
2) Impact of COPD exacerbations on lung function decline, QoL and mortality.
3) Impact of pharmacotherapy and escalation from dual bronchodilator to triple therapy on lung function decline, QoL and mortality.

All interventional and noninterventional studies conducted in human populations and published in English were included. No timeframe was specified. All retrieved citations were screened by two independent reviewers, as per predefined eligibility criteria, after removing duplicates. Discrepancies were resolved by a third reviewer by consensus after discussion. Full-text publications were obtained and screened, and those satisfying the inclusion criteria were included for data extraction. After the selection of eligible studies, data were extracted in a predetermined Microsoft Excel spreadsheet. The extracted data were utilised to form baseline assumptions for FEV_1_, SGRQ and mortality.

### Model assumptions

In this model, we considered the impact of two factors on the progression of COPD. First, exacerbations on lung function decline, and second, the effect of pharmacotherapy in reducing the frequency of exacerbations and hence slowing COPD progression, were superimposed on the underlying changes in lung function with time (FEV_1_, mL⋅year^−1^), SGRQ and mortality.

The model included the following assumptions for FEV_1_, SGRQ and mortality (survival) in COPD over the lifetime of the disease (age 40 to 75 years):

1) In the general population (no COPD diagnosis, no respiratory symptoms and nonsmokers), lung function declines at a constant rate, which was used as a benchmark to assess the per cent decline in COPD [1, 10]. Generally, the downward trajectory of FEV_1_ in COPD patients is steeper than the regular annual lung function decline in the healthy population.
2) FEV_1_ declines at different rates in patients with COPD as the disease progresses over time. The decline in lung function is more rapid with less airflow obstruction (Global Initiative for Chronic Obstructive Lung Disease (GOLD) grades 1 and 2), while it is less rapid with greater airflow obstruction (GOLD grades 3 and 4) [11, 12]. Pharmacotherapy reduces the rate of lung function decline by reducing the frequency of exacerbations.
3) Exacerbations accelerate lung function decline in COPD. Based on published data, the impact of exacerbations was simulated as 1) a progressive increase in exacerbation frequency (0.5→1.0→2.0→3.0 exacerbations per year) and 2) an additional decline of 200 mL in lung function (including a reversible decline of 175.5 mL and a permanent loss of 24.5 mL) with each exacerbation [13–16].
4) SGRQ was used to assess health-related QoL (HRQoL) [17], with higher scores on the questionnaire corresponding to worse QoL in patients [18]. In healthy populations (no COPD), the mean SGRQ score was assigned a value of 6.0, which was used as the baseline value [17]. SGRQ has been reported to change by 0.04 per mL drop in FEV_1_ in COPD [18]. However, preliminary modelling showed that this value was an overestimate considering that we were modelling moderate, rather than severe, COPD; therefore, the final model assumed a 0.02-point increase in SGRQ per mL decline in FEV_1_.
5) Moderate exacerbations increase the probability of death in the following year by 1.8 times the death rate for the matched general population [19, 20].

In the current study, we simulated the impact of pharmacotherapy and escalation from dual bronchodilator to triple therapy on decline in FEV_1_, SGRQ and mortality with 1) long-acting muscarinic antagonist (LAMA)/long-acting β2-agonist (LABA) dual therapy initiated at the age of 45 and continued until the age of 75; and 2) ICS/LAMA/LABA triple therapy initiated at the age of 50, as an escalation from LAMA/LABA dual therapy initiated at the age of 45, with triple therapy continued until the age of 75. This pharmacotherapy usage pattern assumed that a delay often happens in the initiation of dual bronchodilators and that the escalation to triple therapy happens after a few years of dual bronchodilators.

### Model outputs

The following treatment scenarios were investigated in this study:

- No pharmacotherapy initiated between age 40–75 years (designated as the “No pharmacotherapy” group).
- No pharmacotherapy before age 45, LAMA/LABA from age 45–75 (designated as the “LAMA/LABA only” group).
- No pharmacotherapy before age 45, LAMA/LABA from age 45, escalation to ICS/LAMA/LABA at age 50 until age 75 (designated as the “Escalation to triple” group).

The model end-points were changes in FEV_1_, SGRQ and probability of survival over the age range of 40–75 years, with respect to the above treatment scenarios. Data summaries, graphical output, calculations and simulations were produced in Microsoft Excel.

### Sensitivity analysis

We conducted a sensitivity analysis of our findings to understand whether they would also be applicable to a milder COPD population. Therefore, a COPD population experiencing a less-progressive exacerbation frequency of 0.5⋅year^−1^ (one exacerbation every 2 years) from age 40 and then 1.0⋅year^−1^ from age 50 was considered. Treatment scenarios included 1) no therapy between age 40–75 years; 2) no therapy from age 40–55 then LAMA/LABA from age 55; and 3) no therapy from age 40–55 then LAMA/LABA from age 55–60 followed by an escalation to triple therapy from age 60 onwards. The same end-points as the main analysis were investigated.

## Results

### Literature search and summary of extracted data

A total of 44 studies were identified for the development of assumptions for QoL and mortality (supplementary figure S1). The data showed that mortality in patients with COPD receiving triple therapy was lower compared with those receiving dual bronchodilator therapy and mortality for those receiving dual bronchodilator therapy was lower than in those receiving no pharmacotherapy or monotherapy [20]. SGRQ scores correlated with lung function (increasing with lung function decline and decreasing with improvement in lung function) [21, 22]. SGRQ score reduced more rapidly in patients receiving dual bronchodilator or triple therapies, whereas it increased in patients on no pharmacotherapy [7]. All data regarding the assumptions for exacerbations and lung function decline were utilised from the previous analysis [10].

### Decline in lung function (FEV_1_)

**FIGURE 1:**
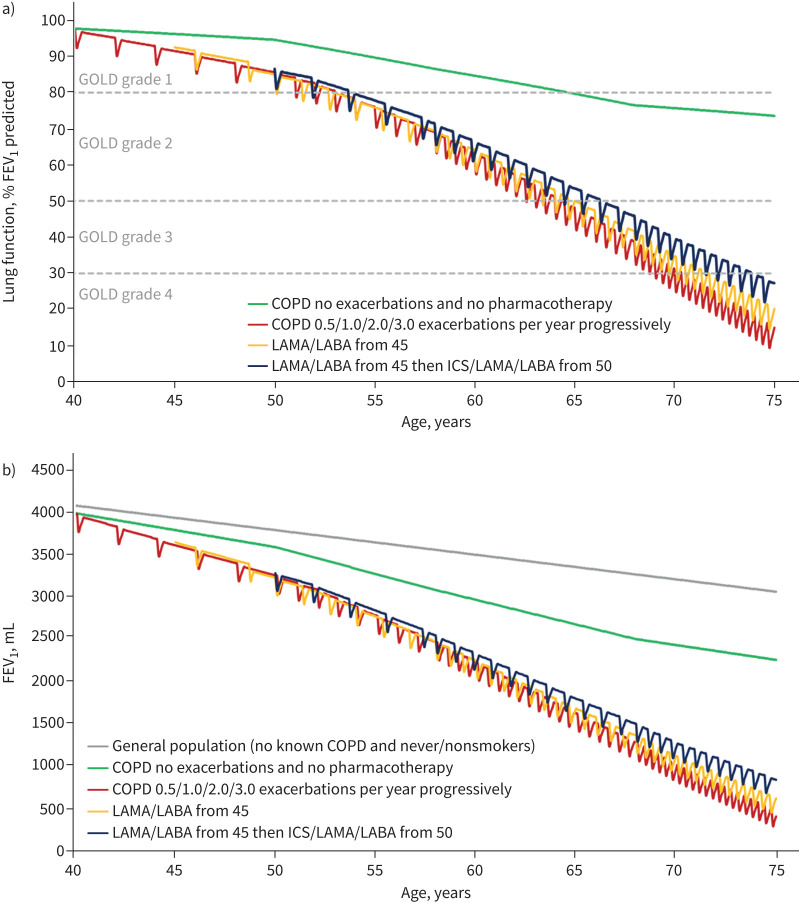
Simulation model output for decline in lung function and impact of initiating dual therapy at age 45 years and escalation to triple therapy at age 50 years, measured as a) forced expiratory volume in 1 s (FEV_1_) % predicted and b) FEV_1_, mL. GOLD: Global Initiative for Chronic Obstructive Lung Disease; LAMA: long-acting muscarinic antagonist; LABA: long-acting β2-agonist; ICS: inhaled corticosteroid.

The model-simulated rate of decline in lung function was expressed as FEV_1_ % predicted (figure 1a) and absolute volume (FEV_1_ mL; figure 1b) from age 40–75 years in a scenario of increasing exacerbations with disease progression (red line). This decline slowed when dual bronchodilator was initiated at the age of 45 years (yellow line). This decline slowed further when dual bronchodilator was escalated to triple therapy (blue line) at 50 years of age.

Compared with the “No pharmacotherapy” group, the “LAMA/LABA only” group had 159.1 mL preservation of lung function at the age of 75 years. The “Escalation to triple” group preserved 376.5 mL and 217.3 mL of lung function by the age of 75, compared with the “No pharmacotherapy” and “LAMA/LABA only” groups, respectively (table 1).

**TABLE 1:** Forced expiratory volume in 1 s (FEV_1_) outcomes for various treatment scenarios of dual bronchodilator therapy *versus* triple therapy in COPD

Treatment scenario	Age when FEV_1_ first drops <30% pred, years	Total number of exacerbations between age 40 and 75 years (mean per year)	Residual FEV_1_ at age 75 years, mL	Preserved FEV_1_ at age 75 years *versus* no therapy, mL	Preserved FEV_1_ at age 75 years for triple escalation *versus* LAMA/LABA only, mL
**No therapy**	69.1	55 (1.6)	456.5		
**No therapy before age 45 years, LAMA/LABA at age 45 years, no escalation to triple**	70.6	43 (1.2)	615.6	159.1	
**No therapy before age 45 years, LAMA/LABA at age 45 years, escalated to triple at 50 years**	72.2	33 (0.9)	833.0	376.5	217.3

LAMA: long-acting muscarinic antagonist; LABA: long-acting β2-agonist.

### Change in QoL (SGRQ)

**FIGURE 2:**
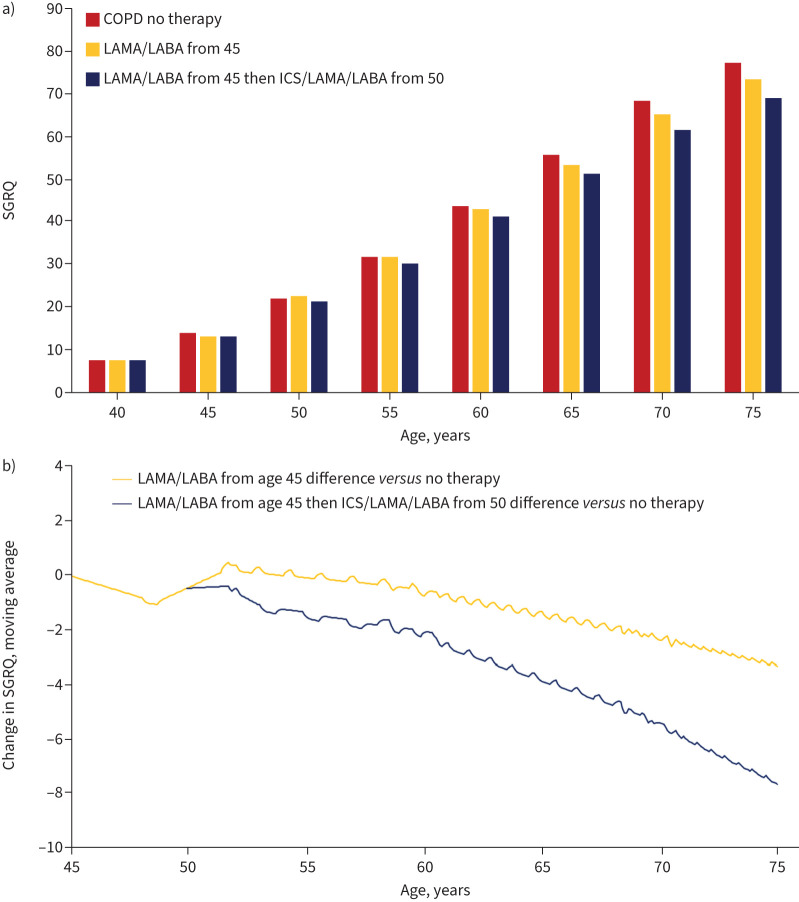
Simulation model output for change in St George's Respiratory Questionnaire (SGRQ) and impact of initiating dual therapy at age 45 years and escalation to triple therapy at age 50 years, measured as a) SGRQ score and b) change in SGRQ. LAMA: long-acting muscarinic antagonist; LABA: long-acting β2-agonist; ICS: inhaled corticosteroid.

The model predicted a decrease in SGRQ score in the “LAMA/LABA only” group (yellow bars) compared with the “No pharmacotherapy” group (red bars; figure 2a). A further decrease was predicted in the “Escalation to triple” group (blue bars; figure 2a).

In the “LAMA/LABA only” group, the SGRQ score decreased by −3.2 and by −7.5 in the “Escalation to triple” group, compared with the “No pharmacotherapy” group (figure 2b and table 2).

**TABLE 2:** St George's Respiratory Questionnaire (SGRQ) outcomes for various treatment scenarios of dual *versus* triple therapy in COPD

Treatment scenario	SGRQ 3-year mean at age 65–68	SGRQ 8-year mean at age 65–73	SGRQ at age 65	SGRQ at age 68	SGRQ at age 73	SGRQ at age 75	SGRQ difference *versus* no therapy at age 75 (mean over previous year)	SGRQ difference *versus* LAMA/LABA at age 75 (mean over previous year)
**No therapy**	57.9	64.3	53.1	60.0	71.8	77.1		
**No therapy before age 45, LAMA/LABA at age 45, no escalation to triple**	55.9	61.8	51.9	58.2	68.9	73.0	−3.2 (−3.7)	
**No therapy before age 45, LAMA/LABA at age 45, escalation to triple at age 50**	53.1	58.5	49.5	55.4	65.2	68.6	−7.5 (−8.5)	−4.4 (−4.8)

LAMA: long-acting muscarinic antagonist; LABA: long-acting β2-agonist.

### All-cause mortality in COPD patients

**FIGURE 3:**
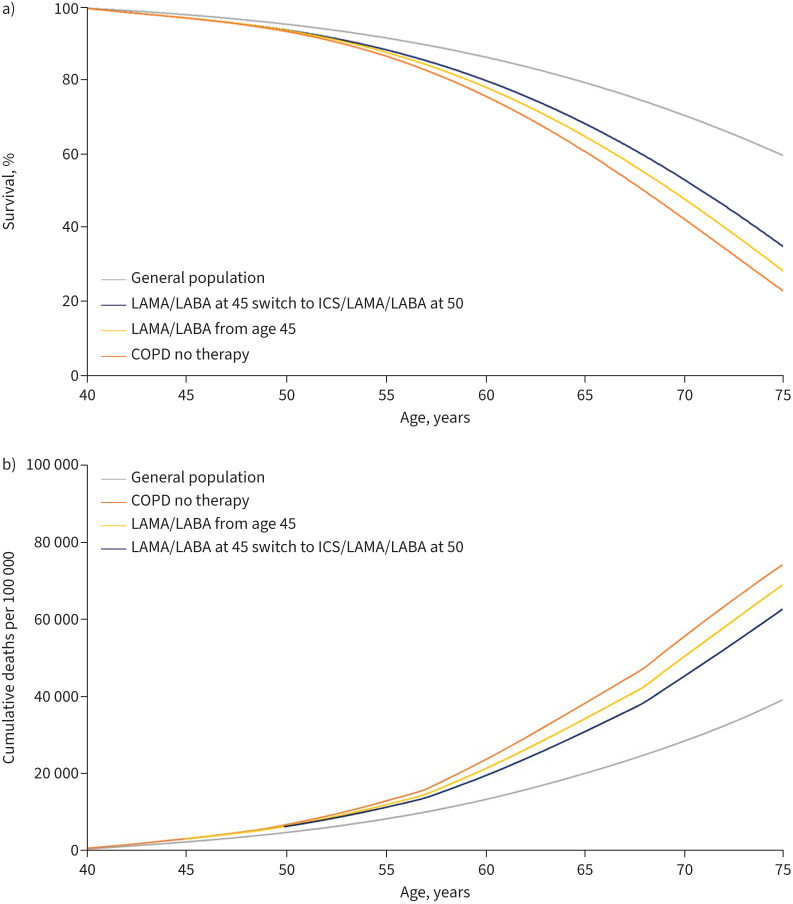
Simulation model output for increase in survival and impact of initiating dual therapy at age 45 years and escalation to triple therapy at age 50 years, expressed as a) % probability of survival and b) cumulative deaths per 100 000. LAMA: long-acting muscarinic antagonist; LABA: long-acting β2-agonist; ICS: inhaled corticosteroid.

The model predicted improved survival (expressed as % probability of survival for 1 year) in the “LAMA/LABA only” group (yellow line; figure 3a), compared with the “No pharmacotherapy” group (red line; figure 3a). Survival improved further in the “Escalation to triple” group (blue line; figure 3a) from age 40–75 years.

Compared with the “No pharmacotherapy” group, the “LAMA/LABA only” group showed a decrease in mortality of 5.4% and the “Escalation to triple” group showed a decrease of 12.0%, resulting in an additional decrease in mortality of 6.6% in the “Escalation to triple” group (table 3 and figure 3b).

**TABLE 3:** Mortality outcomes for various treatment scenarios of dual bronchodilator therapy *versus* triple therapy in COPD

Age range, years	No therapy	LAMA/LABA from age 45	Difference: no therapy *versus* LAMA/LABA from age 45	LAMA/LABA from age 45 then ICS/LAMA/LABA from age 50	Difference: no therapy *versus* LAMA/LABA from age 45 then ICS/LAMA/LABA from age 50	Difference: LAMA/LABA from age 45 *versus* LAMA/LABA from age 45 then ICS/LAMA/LABA from age 50
**65–73**	28 868 (49.4)	27 441 (43.9)	1472 (5.4)	24 759 (37.6)	4109 (11.7)	2682 (6.3)
**65–68**	9225 (15.8)	8413 (13.5)	812 (2.3)	7597 (11.5)	1628 (4.2)	816 (1.9)
**40–75**	73 807 (76.8)	68 573 (71.4)	5235 (5.4)	62 278 (64.8)	11 529 (12.0)	6295 (6.5)

Data are presented as deaths per 100 000 (%). LAMA: long-acting muscarinic antagonist; LABA: long-acting β2-agonist; ICS: inhaled corticosteroid.

The sensitivity analysis demonstrated a similar pattern of results to the baseline model with a smaller decline in FEV_1_ in the “Escalation to triple” group, compared with the other two groups (supplementary figures S2 and S3 and supplementary table S1). Similar patterns were observed for SGRQ (supplementary figures S4 and S5 and supplementary table S2) and mortality (supplementary figures S6 and S7 and supplementary table S3).

## Discussion

This modelling analysis simulated the disease progression in patients with COPD between ages 40 and 75 years and how the initiation of dual bronchodilator therapy at 45 years of age and escalation to triple therapy at 50 years of age could have potential benefits in terms of slowing down lung function decline, improving QoL and improving survival in patients with COPD. The benefits were predicted to be greater when patients escalated from dual bronchodilator to triple therapy, compared with continuing dual bronchodilator therapy. In our previous DEPICT analysis, we reported the long-term benefit of dual bronchodilator or triple therapy on lung function decline when initiated at 40, 55 and 65 years of age, but did not evaluate the impact of escalation to triple therapy from dual bronchodilator therapy neither did we investigate the impact on QoL or survival [10].

The disease course of COPD is often considered to start after 35 years of age, although in many cases, it might even have a much earlier onset. Diagnosis, however, typically occurs much later, or even following a COPD-related hospitalisation, resulting in delayed treatment [23–25]. Studies have shown that COPD is underdiagnosed and often not diagnosed until an exacerbation occurs or there is already a significant decline in lung function [1, 23–25]. According to the GOLD 2024 recommendations, treatment, in most cases, is initiated with dual bronchodilator therapy and may be escalated to triple therapy (which may occur years after bronchodilator therapy) when the criteria for exacerbations are met [1]. This pattern of practice was also reported in a Delphi survey conducted with experts from 16 countries [26]. However, exacerbations themselves are prone to be under-reported due to various factors, although such unreported exacerbations have similar or worse outcomes due to inadequate treatment [27]. As COPD is a progressive disease, it is imperative that the disease is promptly diagnosed and that therapy is initiated promptly to prevent rapid health status decline and reduce the risk exacerbations. It is also important to optimise the treatment in patients with rapidly progressing disease to limit adverse outcomes. Hence, in this analysis, we have focussed on the potential benefits of early initiation and escalation of treatment in patients with COPD, while considering the patterns of routine clinical practice.

Clinical studies report that lung function decline accelerates with increasing frequency of exacerbations [15, 28–30]. Pharmacotherapy aims to control the symptoms, reduce the frequency and severity of exacerbations, and thereby reduce lung function decline in COPD. A systematic review analysing data from approximately 33 000 patients showed that treatment of COPD resulted in a reduction of 5 mL·year^−1^ in lung function decline, compared with untreated COPD [31]. In our earlier DEPICT study, it was observed that early treatment might preserve lung function [10]. In the present analysis, we showed that lung function decline was attenuated when treatment was escalated from dual bronchodilator to triple therapy. However, pharmacotherapy may also improve lung function through other mechanisms not included in our model; hence, our study may underestimate the benefits of early pharmacotherapy on long-term disease progression.

Declining lung function and the occurrence of exacerbation events are significantly correlated with impairment in HRQoL. A baseline elevated SGRQ score is a significant predictor of exacerbations, hospitalisation and death [32]. A QoL worsening in the initial year of follow-up more strongly predicts 10-year mortality by any cause [33]. A decline in QoL often prompts a patient to seek advice, diagnosis and treatment, while a good QoL is an important treatment goal [34]. Studies have reported that early pharmacological intervention is associated with improved QoL [9]. In our analysis, a decrease in SGRQ score with dual bronchodilator therapy at the age of 45 years was observed (−3.2) and an additional decrease was observed when escalated to triple therapy (−4.4), indicating a possibility of the disease-modifying effects of ICS-based therapy. This is in line with clinical studies where triple therapy showed a significant decrease in SGRQ compared with ICS/LABA and LAMA/LABA [7, 35].

An increasing number and severity of exacerbations were shown to be associated with increased all-cause mortality, as well as COPD-related mortality [36]. Müllerova *et al*. [32] found that the risk of death was increased more than twice among patients with the poorest health status (14.3%) compared with those with the best (6.8%). Inhaled triple therapy not only has positive effects on lung physiology, symptoms and exacerbations but also shows a reduction in mortality [37]. In the phase 3 IMPACT trial, triple therapy reduced mortality compared with dual bronchodilator therapy (2.36% *versus* 3.19% for triple and dual bronchodilator therapy, respectively) [38].

A limitation of this analysis is that exacerbations were modelled as occurring at regular intervals, whereas, in real life, patterns of exacerbations are likely to be random. Data from short-to-medium clinical trials were used to build the model that described a disease course over 30 years due to the unavailability of long-term interventional data. The model was built on data from multiple studies and used mean estimates for a broader approach, instead of data from a single trial. Though mean estimates are presented in this analysis, there might be fast decliners who would do worse than our estimates.

We have not included multiple covariates and their plausible values. This approach over a long period would not be feasible as the events would occur at various time points (each patient developing comorbidities at different times). This approach would also require significant data, which are currently unavailable. Hence, we chose to model an average or typical patient outcome over time. This approach, however, would not influence the overall outcome, as we modelled the average outcomes over a long period of time.

We considered all LABA/LAMA and ICS/LABA/LAMA combinations to be equally effective. However, this might not be the case in the real world. While differences are likely to be minor, some head-to-head studies indicate differences in FEV_1_ improvements [39]. It is possible that such differences in trough FEV_1_ improvements could also have small, but in the long-term potentially meaningful, effects on the rate of FEV_1_ decline. The same could apply to differences in the ICS component. However, accounting for such differences is beyond the scope of the current study; hence, the outcomes should be interpreted with clinical observations taken into consideration.

Another limitation of our study is that the inputs for increase in FEV_1_, improvement in SGRQ scores and mortality benefit from different pharmacotherapies represent average values across COPD age groups, and subgroup analyses, based on eosinophil blood count and comorbid conditions, *etc.* were not considered. However, the aim of our study was to assess the effect of pharmacotherapy on a broad COPD population rather than a particular subgroup. Subgroup analysis could be an opportunity for future research. Also, in clinical practice, patient pathways to triple therapy may differ but this was not accounted for in our analysis. Further, we assumed a continuous progression of COPD, whereas it is possible that different subgroups or phenotypes of COPD patients show a variable natural history [40]. For this reason, we also conducted a sensitivity analysis of our findings to understand whether they are likely to be applicable to a COPD population experiencing fewer exacerbations. Additionally, we did not consider the safety profile of the inhalers or scenarios with low adherence and compliance with the treatment regimen. It should be noted that safety was not considered in this modelling exercise and that high levels of compliance and adherence, as typically found in COPD clinical trials, were assumed to apply the groups under consideration (dual (with and without switching) as well as the triple therapy group). The adverse event profile has already been modelled for pneumonia in patients receiving ICS-based triple therapy in a previous study [41]. The potential adverse effects of early treatment escalation from dual to triple therapy and how it affects the compliance and adherence over a lifetime horizon of a patient is a potential topic for further research.

Another limitation is that we evaluated the effects of pharmacotherapy on FEV_1_, SGRQ and mortality, which may have introduced an unintentional bias in favour of an ICS-containing regimen. However, all these parameters are well established measures of treatment efficacy and, therefore, we considered them in the current study.

The RETHINC trial [42], explored the efficacy of dual bronchodilation in patients who had an FEV_1_/forced vital capacity (FVC) ratio of >0.7, and showed negative results on its primary objective, a decrease in respiratory symptoms. The methodological issues for RETHINC included the short follow-up and reduced sample size (535 participants) [43]. The present model is for patients with FEV_1_/FVC<0.7, and it is well known that lung function decline is more rapid in mild COPD and initiation of treatment in mild COPD also holds merit. DEPICT-2 concluded a significant benefit in FEV_1_ % predicted of 2.48 (95% CI 1.49–3.47), and also in inspiratory capacity, which requires further research and perhaps more trials.

To our knowledge, this is the only study so far to show the potential benefits of early initiation of pharmacotherapy and escalation from dual bronchodilator to triple therapy on lung function, QoL and mortality in patients with COPD. The assumptions in this study were derived from published literature and the scenarios were based on common clinical practice. Input data included a wide variation in patient population, including those with various comorbidities, and is an average of data from multiple interventional and observational studies, which takes broad variations in COPD patients into account. The model addressed a key question about the long-term impact of pharmacotherapy on COPD, which is a lifelong disease. The results highlight the need for early diagnosis and rapid escalation to triple therapy in appropriate patients, to maximise potential long-term benefits.

### Conclusion

This modelling study concludes that early initiation and early escalation of treatment from dual bronchodilator to triple therapy in patients at high risk of COPD exacerbations may be associated with long-term improvements in key outcomes such as lung function, QoL and mortality.

## Supplementary material

10.1183/23120541.00438-2024.Supp1 **Please note:** supplementary material is not edited by the Editorial Office, and is uploaded as it has been supplied by the author.
Supplementary material 00438-2024.SUPPLEMENT

## Data Availability

The datasets generated during and/or analysed during the current study are available from the sponsor on reasonable request.
