# Supplementary material for: Delaying disease progression in COPD with early escalation to triple therapy: a modelling study (DEPICT-2)
Source: ERJ Open Res. 2025 Apr 7;11(2):00438-2024. doi: 10.1183/23120541.00438-2024 (PMC11973711; doi:10.1183/23120541.00438-2024)
Supplement: Supplementary file 1 [file 00438-2024.SUPPLEMENT.pdf]

DEPICT-2 Supplement

Figure S1: PRIMA Flowchart for targeted literature review

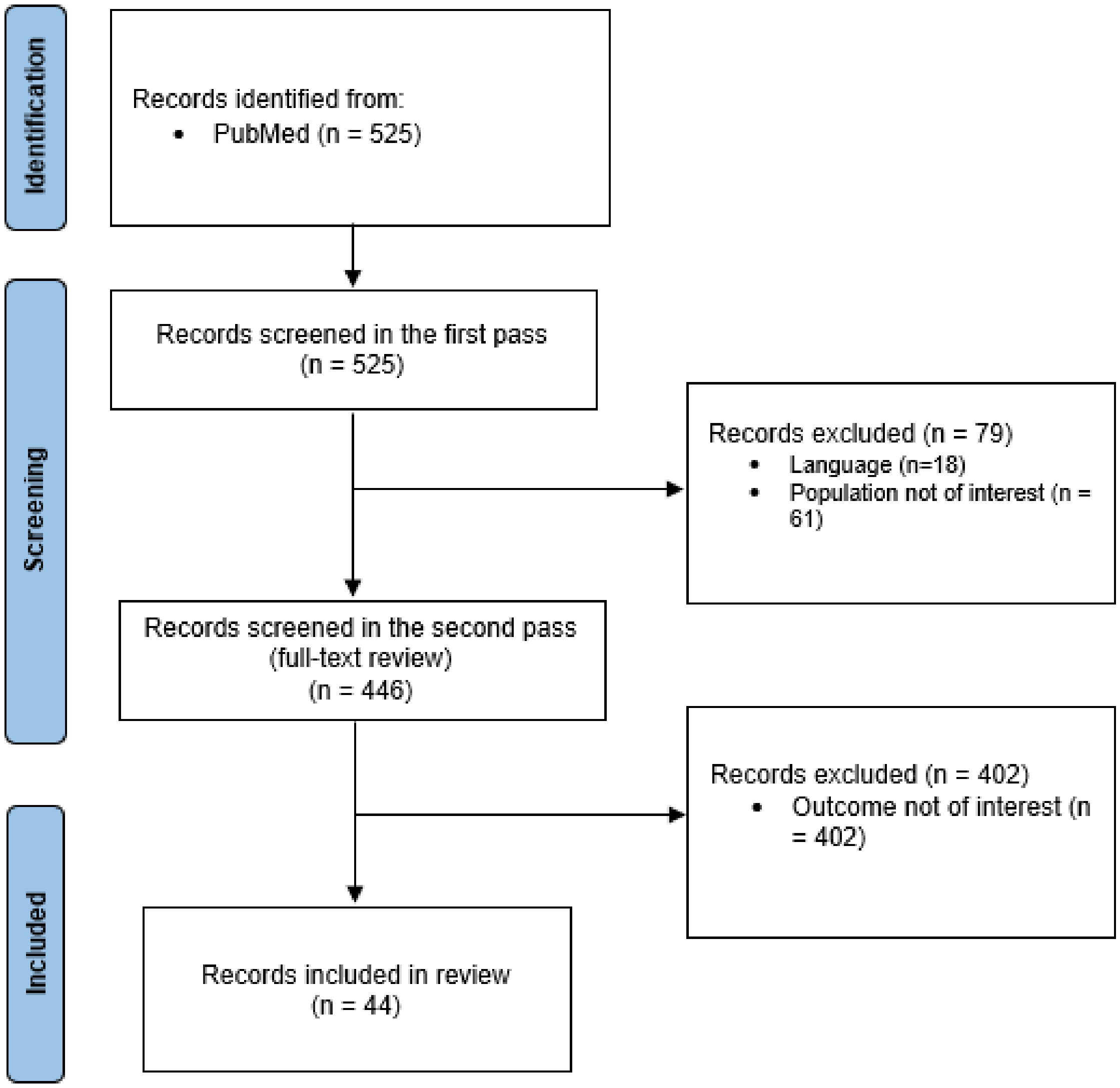

Figure S2: Sensitivity analysis: Modelled outcomes for **FEV_1 % predicted** for various treatment scenarios in a COPD population experiencing a progressive exacerbation frequency (0.5 → 1.0 exacerbations/year)

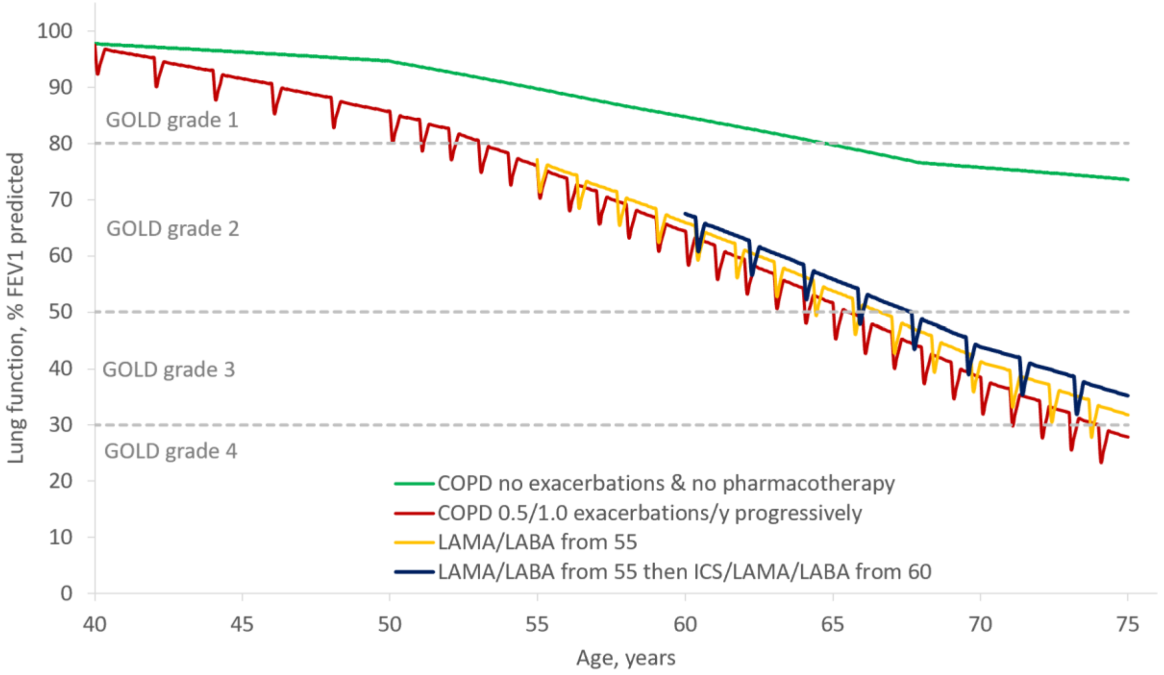

Figure S3: Sensitivity analysis: Modelled outcomes for **FEV_1 (mL)** for various treatment scenarios in a COPD population experiencing a progressive exacerbation frequency (0.5 → 1.0 exacerbations/year)

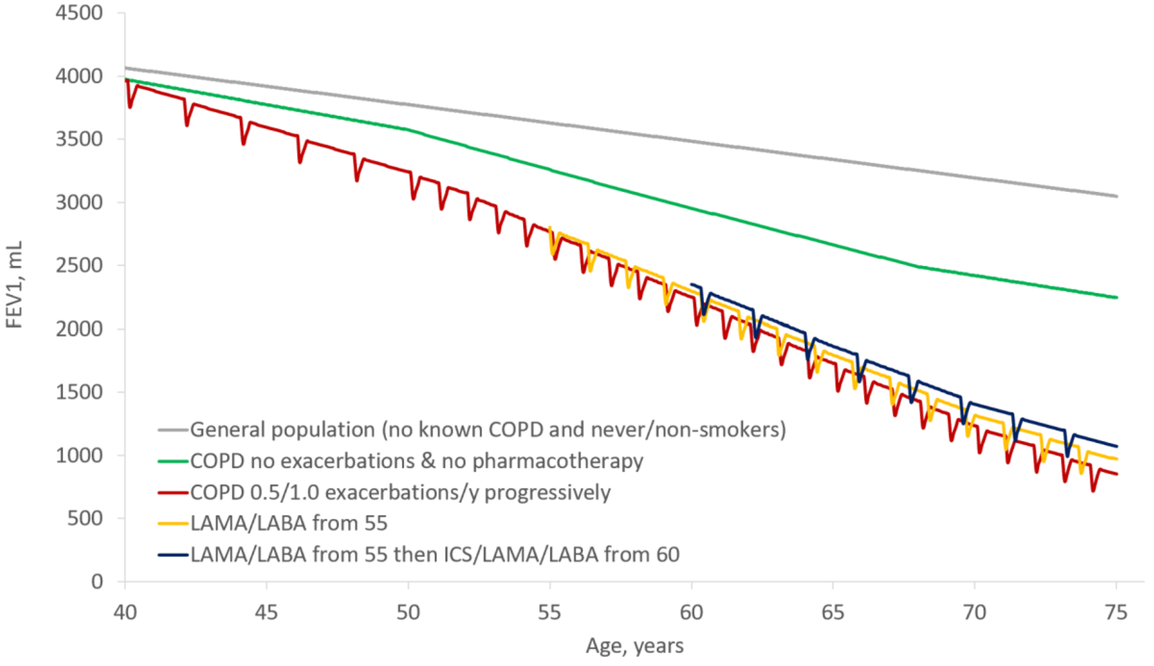

Figure S4: Sensitivity analysis: Modelled outcomes for **SGRQ** for various treatment scenarios in a COPD population experiencing a progressive exacerbation frequency (0.5 → 1.0 exacerbations/year)

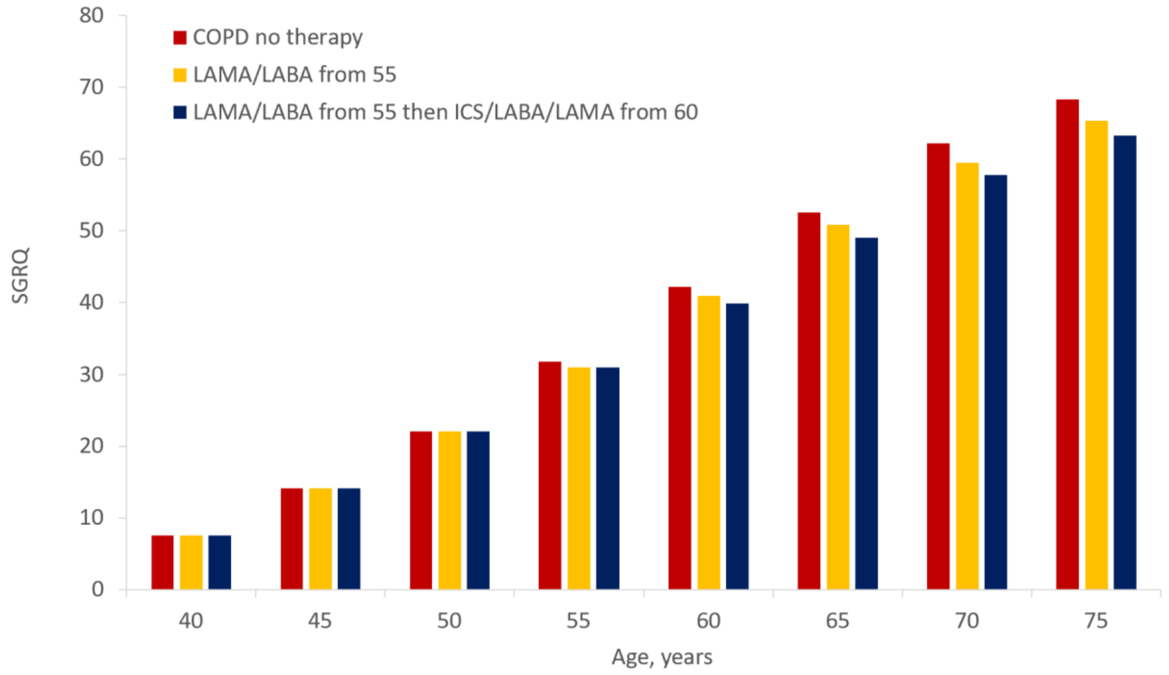

Figure S5: Sensitivity analysis: Modelled outcomes for **average change in SGRQ versus no therapy** for various treatment scenarios in a COPD population experiencing a progressive exacerbation frequency (0.5 → 1.0 exacerbations/year)

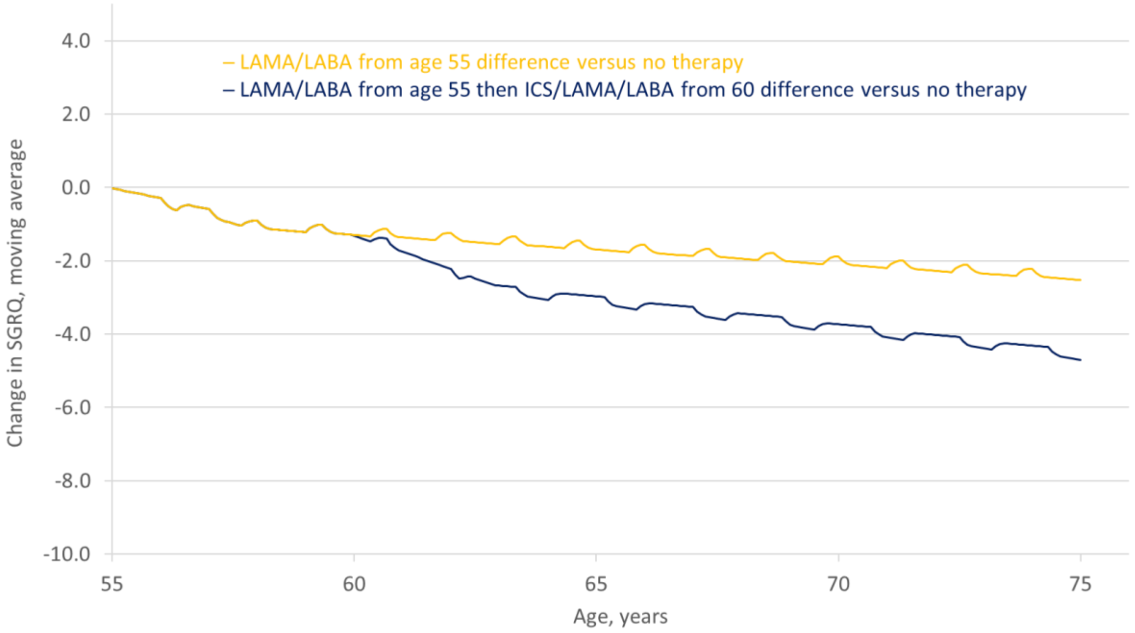

Figure S6: Sensitivity analysis: Modelled outcomes for **Survival (%, probability of survival for one year)** for various treatment scenarios in a COPD population experiencing a progressive exacerbation frequency (0.5 → 1.0 exacerbations/year)

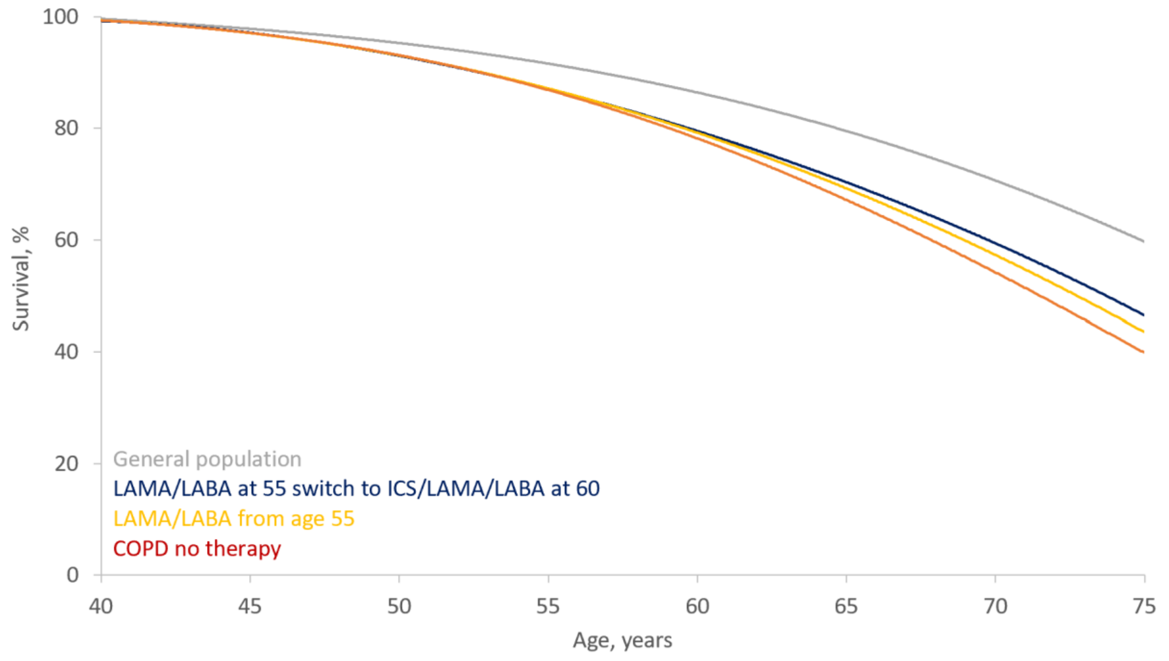

Figure S7: Sensitivity analysis: Modelled outcomes for **mortality (cumulative deaths per 100,000)** for various treatment scenarios in a COPD population experiencing a progressive exacerbation frequency (0.5 → 1.0 exacerbations/year)

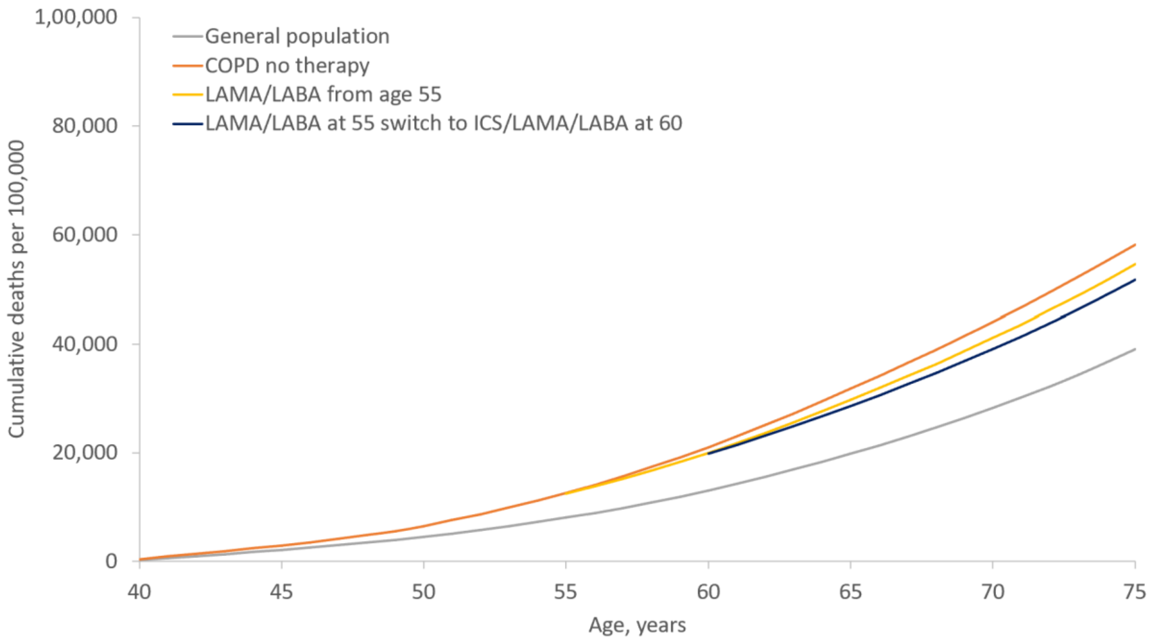

Table S1: Supplementary table: Sensitivity analysis: Dual versus Triple therapy in COPD: FEV1 outcomes for various treatment scenarios

**Treatment scenario**	**Age FEV_1 first drops below 30%**	**Total number of exacerbations between age**	**Residual FEV_1 at**	**Preserved FEV_1 at age 75**	**Preserved FEV_1 at**

	predicted<br><br>Years	40 and 75<br><br>(average per year)	age 75<br><br>mL	versus no therapy<br><br>mL	age 75<br><br>for triple switch versus LAMA/LABA only<br><br>mL
No therapy	72.08	30<br><br>(0.86)	848.5	-	-
No therapy before age 55, LAMA/LABA at age 55,<br><br>**No switch** to triple	73.75	25<br><br>(0.71)	970.2	121.7	-
No therapy before age 55, LAMA/LABA at age 55,<br><br>**Switch** to triple at age 60	>75*	22<br><br>(0.63)	1073.2	224.7	103.0
* FEV_1 = 35.2% predicted at age 75 years					

Table S2: Supplementary table: Sensitivity analysis: Dual versus Triple therapy in COPD: SGRQ outcomes for various treatment scenarios

**Treatment scenario**	**SGRQ**<br>**3-year average age**<br>**65-68**	**SGRQ**<br>**8-year average**<br>**age**<br>**65-73**	**SGRQ**<br>**at age 65**	**SGRQ**<br>**at age 68**	**SGRQ**<br>**at age 73**	**SGRQ**<br>**at age 75**	**SGRQ difference versus**<br>**no therapy**<br>**at age 75**<br>**(Average over previous year)**	**SGRQ difference versus LAMA/LABA**<br>**at age 75**<br>**(Average over previous year)**
No therapy	54.5	59.2	50.8	56.8	65.3	68.3	-	-
No therapy before age 55, LAMA/LABA at age 55,<br>**No switch** to triple	52.6	57.1	49.4	55.0	63.2	65.9	-2.43<br>(-3.04)	-
No therapy before age 55, LAMA/LABA at age 55,<br>**Switch** to triple at age 60	51.1	55.3	48.0	53.6	61.1	63.8	-4.49<br>(-5.10)	-2.06<br>(-2.06)

Table S3: Supplementary table: Sensitivity analysis: Modelled outcomes for COPD mortality, deaths/100,000 (%) for various treatment scenarios in a COPD population experiencing a progressive exacerbation frequency (0.5→1.0 exacerbations/year)

Age range Years	No therapy	LAMA/LABA from age 55	Difference: No therapy versus LAMA/LABA from age 55	LAMA/LABA from age 55 then ICS/LAMA/LABA from age 60	Difference: No therapy versus LAMA/LABA from age 55 then ICS/LAMA/LABA from age 60	Difference: LAMA/LABA from age 55 versus LAMA/LABA from age 55 then ICS/LAMA/LABA from age 60
**65-73**	20475 (31.7%)	19106 (28.7%)	1369 (3.0%)	17684 (26.1%)	2790 (5.6%)	1421 (2.6%)
**65-68**	7187 (11.1%)	6618 (9.9%)	569 (1.2%)	6059 (8.9%)	1128 (2.2%)	559 (1.0%)
**40-75**	57884 (60.2%)	54248 (56.4%)	3636 (3.8%)	51428 (53.5%)	6456 (6.7%)	2820 (2.9%)

Total and % deaths for each age range in the remaining population assuming 96,425 of 100,000 (96.4%) were alive at age 40.

Model assumes corresponding death rates in general population are: 65-73 = 18.9%, 65-68 = 6.3% and 40-75 = 40.3%)
